# Identification of Latent Diagnostic Biomarkers and Biological Pathways in Dermatomyositis Based on WGCNA

**DOI:** 10.1155/2021/1920111

**Published:** 2021-12-30

**Authors:** Shaxi Ouyang, Yifang Liu, Changjuan Xiao, Qinghua Zeng, Xun Luo, Xiaofang Hu, Shuoshan Xie

**Affiliations:** ^1^Nephrology Department and Laboratory of Kidney Disease, Hunan Provincial People's Hospital, The First Affiliated Hospital of Hunan Normal University, Changsha, China; ^2^Changsha Clinical Research Center for Kidney Disease, Changsha, China; ^3^Hunan Clinical Research Center for Chronic Kidney Disease, Changsha, China; ^4^Department of Clinical Medicine, School of Medicine, Hunan Normal University, Changsha, Hunan 410013, China

## Abstract

**Introduction:**

Dermatomyositis (DM) is a chronic autoimmune disease of predominantly lymphocytic infiltration mainly involving the transverse muscle. Its pathogenesis is remaining unknown. This research is designed to probe the latent pathogenesis of dermatomyositis, identify potential biomarkers, and reveal the pathogenesis of dermatomyositis through information biology analysis of gene chips.

**Methods:**

In this study, we utilised the GSE14287 and GSE11971 datasets rooted in the Gene Expression Omnibus (GEO) databank, which included a total of 62 DM samples and 9 normal samples. The datasets were combined, and the differentially expressed gene sets were subjected to weighted gene coexpression network analysis, and the hub gene was screened using a protein interaction network from genes in modules highly correlated with dermatomyositis progression.

**Results:**

A total of 3 key genes—myxovirus resistance-2 (MX2), oligoadenylate synthetase 1 (OAS1), and oligoadenylate synthetase 2 (OAS2)—were identified in combination with cell line samples, and the expressions of the 3 genes were verified separately. The results showed that MX2, OAS1, and OAS2 were highly expressed in LPS-treated cell lines compared to normal cell lines. The results of pathway enrichment analysis of the genes indicated that all 3 genes were enriched in the cytosolic DNA signalling and cytokine and cytokine receptor interaction signalling pathways; the results of functional enrichment analysis showed that all 3 were enriched in interferon-*α* response and interferon-*γ* response functions.

**Conclusions:**

This is important for the study of the pathogenesis and objective treatment of dermatomyositis and provides important reference information for the targeted therapy of dermatomyositis.

## 1. Introduction

Dermatomyositis (DM) is a relatively rare idiopathic multisystem inflammatory disease with a small incidence of just 1 in 100,000 and a significantly higher incidence in women than in men among adult patients [1–3]. Dermatomyositis is difficult to diagnose accurately without the presence of a characteristic dermatologic or myopathic condition [4]. Patients with typical dermatomyositis usually have a very abnormal skin surface, accompanied by progressive, symmetrical proximal muscle weakness. 30–50% of patients develop cutaneous disease 3–6 months before the appearance of myositis, while approximately 10% develop muscle symptoms before the appearance of cutaneous disease [5, 6]. About 20% of DM cases are classified as clinically amyopathic dermatomyositis (CADM), which is strongly associated with acute interstitial lung lesions, interstitial fibrosis, and has a very high mortality rate. In a study of 291 patients with CADM [7], 70% were diagnosed with nonamyopathic dermatomyositis and 13% with amyopathic dermatomyositis. Clinically, complete remission of cutaneous lesions is very difficult to achieve, which reflects the lack of understanding of the pathogenesis of dermatomyositis cutaneous lesions [8]. As the traditional view is that the pathogenesis of autoimmune diseases is mainly due to excessive activation of effector T cells, glucocorticoids and immunosuppressive drugs are currently the main treatments for dermatomyositis [9]. The use of these drugs weakens the immunity of humoral and cellular immunity and also decreases the immune function of intrinsic immunity such as macrophages and NK cells, decreasing the body's resistance to pathogens and increasing the incidence of opportunistic infections, such as the Epstein-Barr Virus (EBV) and cytomegalovirus (CMV) blood disorders [10].

In addition, common histopathologic factors of DM skin lesions, often including vacuolar perivascular inflammation, interface dermatitis, increased skin mucin, and keratinised abnormal keratin-forming cells [11, 12], are also seen in cutaneous lupus erythematosus (CLE) lesions. This can make it more difficult to differentiate a rash associated with DM from one involving CLE, which makes the diagnosis of DM more difficult. In addition, dermatomyositis is closely associated with several types of cancer. Most patients have cancer within 1 year after the diagnosis of dermatomyositis, and thus, dermatomyositis is considered to be a paraneoplastic condition [13]. Therefore, it is urgent to find new diagnostic concepts and therapeutic approaches for dermatomyositis, and studies related to the identification of mRNA biomarkers for dermatomyositis are needed.

This manuscript is designed to probe the possible molecular mechanisms of DM. By identifying effective biomarkers through microarray analysis and then validating them through in vitro experiments, the pathogenesis of DM can be elucidated as well as the search for targeted therapies for DM.

## 2. Methods

### 2.1. Data Sources

The dermatomyositis gene datasets GSE142807 [8] (43 DM samples and 5 normal samples) and GSE11971 [14] (19 DM samples and 4 normal samples) were rooted in the Gene Expression Omnibus (GEO) databank, and the original document were processed and explained with the R package “affy” in a bioconductor for processing and annotation (http://www.bioconductor.org).

### 2.2. Cell Nurturing and Stimulation of Cells

Human skeletal muscle myoblasts (HSkM) were purchased from ScienCell Research Laboratories, located in USA. The myoblasts were cultured in Dulbecco's modified Eagle medium containing 4.5 mg/mL glucose + MEM 199 (ratio 4 : 1) with 20% fetal bovine serum, 100 IU penicillin, and 100 *μ*g streptomycin. Myogenic cells were cultured in 6-well plates at a concentration of 5 × 104/mL at 37°C in a 5% CO_2_ incubator, and the medium was renewed when the cells were fused to 60%. Lipopolysaccharides (LPS) [15] were used to stimulate myogenic cells to prepare a dermatomyositis cell model, after which the cells were collected for RNA-seq.

### 2.3. Methods for RNA Extraction and Transcript Library Formation

We first extracted total RNA from the cells using TRIzol reagent and then constructed RNA samples by RNA mass spectrometry using a Nanodrop microspectrophotometer. After enrichment of eukaryotic mRNA with polyA tails by magnetic beads with oligo (dT), mRNA was interrupted with buffer. We first synthesized cDNA first strand in the M-MuLV reverse transcriptase system using fragmented mRNA as template and random oligonucleotides as primers and then degraded RNA strand with RNaseH and synthesized cDNA second strand with dNTPs in the DNA polymerase I system. The purified double-stranded cDNA was end-repaired, A-tailed, and sequenced, and the cDNA of about 200 bp was screened with AMPure XP beads, then PCR amplified, and the PCR product was purified again with AMPure XP beads, and finally, the final result was achieved.

### 2.4. Differentially Expressed Gene Screening

Differential analysis of normal and dermatomyositis samples in the GEO dataset was performed using the “limma” package in R v4.0.4; differential analysis of normal and dermatomyositis model cell samples was performed using the DEseq2 package in R v4.0.4. The screening criteria were FDR < 0.05 and log2|FC|≥1.

### 2.5. Weighted Gene Coexpression Network Analysis (WGCNA)

WGCNA was executed using the WGCNA database in R v4.0.4 as follows. The correlation coefficients between pairs of all genes were calculated to construct the gene expression correlation matrix. After this, the correlation coefficients are weighted with power exponents, so that the correlation matrix of the expression is transformed into an adjacency matrix. The topological matrix (TOM) is used to calculate the association between genes, and the adjacency matrix is converted into a topological matrix based on the TOM values. The topological matrix has a prescribed algorithm for node dissimilarity, and the different gene modules are clustered using node dissimilarity. The expression of module eigengene (ME) and gene significance (GS) were calculated to associate different modules with phenotypes.

### 2.6. Protein-Protein Interaction Network (PPI)

The mechanism of protein-protein interactions was established at the online analysis website, Metascape (https://metascape.org/gp/index.html#/main/step1). The MCODE algorithm in Cytoscape was used to extract hub genes and visualise the protein-protein interaction network.

### 2.7. KEGG and GO Enrichment Analyses

We established gene ontology (GO) and Kyoto Encyclopedia of Genes and Genomes (KEGG) pathway analysis of genes in the DAVID 6.8 database (https://david.ncifcrf.gov/). Enrichment results with *p* < 0.05 or FDR < 0.05 would indicate that it is statistically significant.

### 2.8. Gene Set Enrichment Analysis (GSEA)

We wanted to know how gene expression affects the disease and divided the samples into high and low expression series on the basis of the median expression values of the genes. The GSEA tool rooted in the Broad Institute (http://software.broadinstitute.org/gsea/downloads.jsp) was used to analyse the enrichment of KEGG and Hallmark pathways in the high and low expression series. Molecular characterisation was done making the use of the Hallmark gene set database (MsigDB, http://software.broadinstitute.org/gsea/msigdb). These pathways were considered meaningful gene sets when they satisfied |NES|≥1, FDR < 0.25, *p* value < 0.05.

### 2.9. Statistical Analysis

Statistical analyses were performed using R software v4.0.3 (R Foundation for Statistical Computing, Vienna, Austria). One-way ANOVA is taken for the samples with uniform variance, and the nonparametric test is taken for the samples with uneven variance. *P* value of <0.05 was considered statistically significant.

## 3. Results

### 3.1. Differentially Expressed Genes in Dermatomyositis

**Figure 1:**
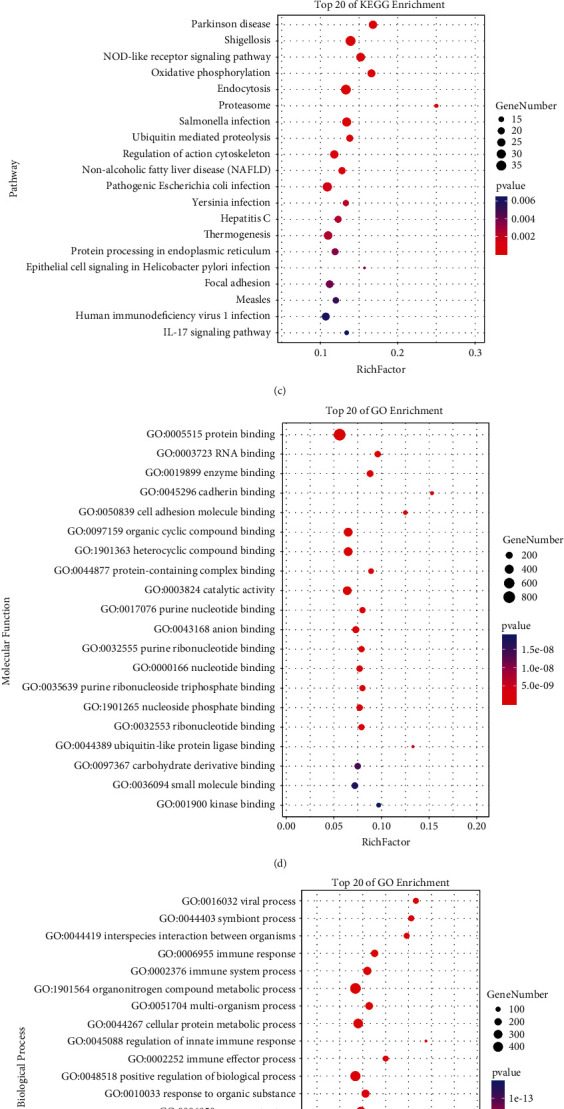
Analysis of differentially expressed genes in GSE14807 and GSE11971 datasets. (a) Volcano plot showing differentially expressed genes in GSE14807. (b) Volcano plot showing differentially expressed genes in GSE11971. (c) Enrichment analysis of KEGG pathway for differential gene ensemble. (d) Enrichment analysis of molecular function for differential gene ensemble. (e) Enrichment analysis of biological processes for differential gene ensemble. (f) Enrichment analysis of cellular components for differential gene ensemble.

The GEO database was used to obtain the DM-related expression datasets GSE142807 (43 DM samples and 5 normal samples) and GSE11971 (19 DM samples and 4 normal samples). After differentially expressed gene screening, all 2746 upregulated genes and 382 downregulated genes were obtained from GSE142807 (Figure 1(a)); all 236 upregulated genes and 663 downregulated genes were obtained in GSE11971. Subsequently, the two differential gene datasets were combined using the ComBat function of the R package “sva” to remove the batch effect, and a total of 925 gene expression matrices were obtained. Functional and pathway enrichment analysis was established, and the results showed that the gene sets were significantly enriched in KEGG pathways, including shigellosis, endocytosis, and Alzheimer's disease (Figure 1(c)). GO functional enrichment analysis significantly enriched the gene set, mainly in protein binding, metabolic processes of organic nitrogen compounds, and intracellular fractions (Figures 1(d)–1(f)).

### 3.2. Analysis of the Weighted Gene Coexpression Network

**Figure 2:**
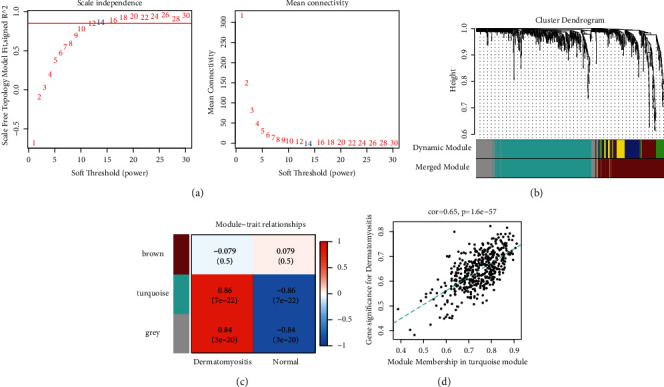
Construction of the weighted coexpression network and identification of key modules. (a) Network topology analysis of soft thresholds. (b) Identification of coexpression modules and cluster dendrogram. (c) Heat map analysis of module gene and clinical phenotype correlations. (d) Turquoise correlation of genes within modules with clinical phenotype data.

The expression information of the combined GSE142807 and GSE11971 differential gene sets were used as input files, and the samples were first hierarchically clustered to eliminate outlier samples. To determine the scale-free network, we set power = 14 as a soft threshold parameter and then constructed a coexpression matrix (Figure 2(a)). The network graph was constructed using dynamic tree cuts and merging similarity modules, so we could obtain 3 groups of 925 genes, and these different sections have been marked with different color notations (Figure 2(b)). Next, the Pearson correlation between different modules and different clinical characteristics was calculated.

The Pearson correlation coefficients of different modules with different clinical features were calculated, and the most relevant module for dermatomyositis was obtained: the turquoise module (Figure 2(c)). The association between the genes in the turquoise module and the clinical phenotypes of dermatomyositis was analysed separately, and the correlation was good, with a significant linear correlation (Figure 2(d)).

### 3.3. Protein-Protein Interaction Network Analysis (PPI)

**Figure 3:**
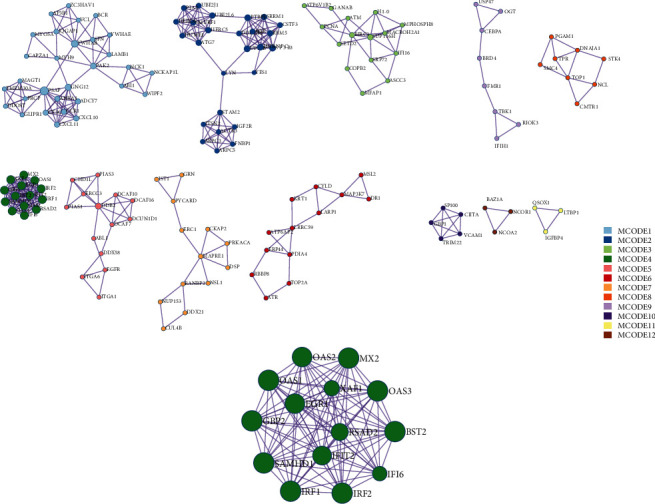
Map of gene interaction network in turquoise module.

Through the online analysis website Metascape, the genes in the turquoise module were analysed to obtain PPI protein network interactions, and the gene information was further visualized and the network was constructed (Figure 3). We use the MCODE plugin in Cytoscape to count the features of each node in the network graph, and the MCODE with the largest score value 4 was selected; genes in MCODE 4 were MX2, GBP2, OAS2, IFI6, IFIT2, BST2, OAS3, OAS1, IRF1, SAMHD1, RSAD2, EGR1, XAF1, and IRF2, which were mainly enriched in the interferon signalling pathway and immune system in the cytokine signalling pathway, as given in Table 1.

### 3.4. Cell Line RNA-Seq Analysis

**Figure 4:**
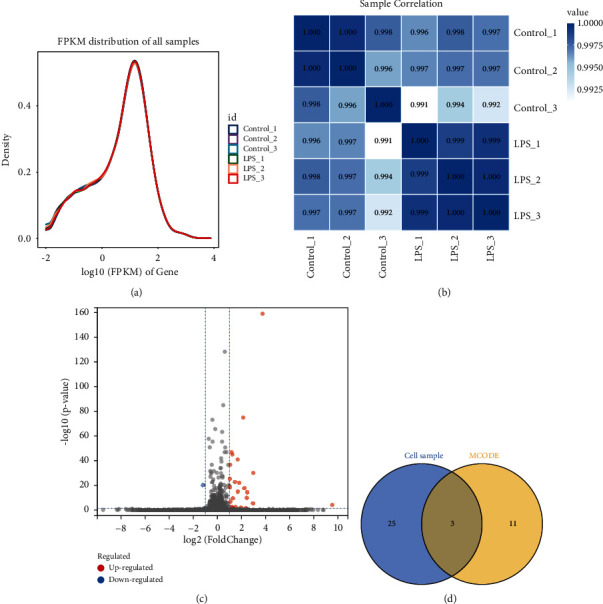
Cell line RNA-seq analysis. (a) Gene expression abundance map. (b) Sample correlation heat map. (c) Volcano map showing differentially expressed genes. (d) Overlap of differentially expressed genes in cell lines with genes in MCODE4.

Based on the FPKM values of each gene in the cell lines, we show the expression distribution of different sample genes or transcripts by an expression distribution map (Figure 4(a)). In general, the gene expression distribution map can be used to assess the differences between samples in the library in terms of building, sequencing, comparison, or quantification. In addition, based on the expression results of each sample, we used PCA analysis and calculated Pearson correlation coefficients between samples to determine the reproducibility between samples and to assist in excluding outliers (Figure 4(b)). The differentially expressed genes in the cell line samples are shown in a volcano plot (Figure 4(c)), and all 29 differentially expressed genes were obtained, including 27 differentially upregulated genes and 2 differentially downregulated genes. By overlaying the differentially expressed genes in the cell line samples with the previous genes in MCODE4, 3 key genes are obtained with the names MX2, OAS1, and OAS2.

### 3.5. Expression Validation of MX2, OAS1, and OAS2

**Figure 5:**
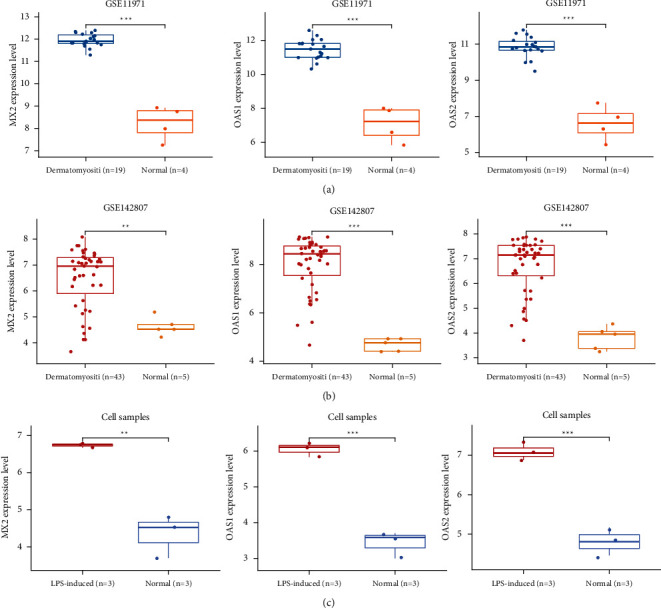
Expression validation of MX2, OAS1, and OAS2. (a) Expression of MX2, OAS1, and OAS2 in the GSE11971 dataset. (b) Expression of MX2, OAS1, and OAS2 in the GSE142807 dataset. (c) Expression of MX2, OAS1, and OAS2 in cell line samples.

The expressions of MX2, OAS1, and OAS2 in the 3 different datasets were compared. The results showed that MX2, OAS1, and OAS2 were significantly upregulated in the GSE11971 and GSE142807 datasets compared to normal samples in dermatomyositis samples (Figures 5(a) and 5(b)). In cell line samples, MX2, OAS1, and OAS2 were highly expressed in LPS-treated cell lines compared to normal cell lines (Figure 5(c)).

### 3.6. Genomic Enrichment Analysis

**Figure 6:**
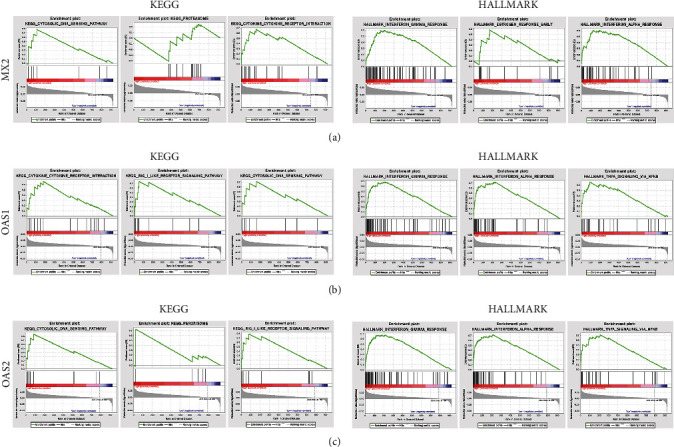
Gene set enrichment analysis. (a) MX2 in KEGG and Hallmark signalling pathway enrichment analyses results. (b) OAS1 in KEGG and Hallmark signalling pathway enrichment analyses results. (c) OSA2 in KEGG and Hallmark signalling pathway enrichment analyses results.

KEGG signalling pathway and Hallmark functional enrichment analyses were performed for each of MX2, OAS1, and OAS2. KEGG signalling pathway analysis showed that all 3 were enriched in the cytosolic DNA signalling and cytokine and cytokine receptor interaction signalling pathways; the results of Hallmark functional enrichment analysis showed that all 3 were enriched in interferon-*α* response and interferon-*γ* response functions (Figure 6).

## 4. Discussion

Dermatomyositis is due to autoimmune connective tissue lesions, which usually include diseases such as autoantibody positivity and immune abnormalities. It has a complex clinical presentation, so there is little hope of a cure [16, 17]. Skin invasion is usually visible in all dermatomyositis subtypes, and skin problems often persist after successful treatment of muscle disease, greatly affecting patients' quality of life [18]. In a prospective cohort study of 74 patients with DM who received systemic therapy, only 38% of patients had remission of skin disease during the 3-year follow-up period [19]. Because many physicians have difficulty recognising dermatomyositis in the absence of muscle invasion, this often leads to misdiagnosis, as well as delays in treatment and initial investigations, and delays or misdiagnosis can increase the risk of cancer in patients [20, 21]. There is therefore a pressing need for appropriate biomarkers to identify dermatomyositis in clinical practice.

In this study, we used a multistep approach to identify differentially expressed genes in DM from microarray data and performed weighted gene coexpression network and protein interaction network analyses. Combined with in vitro experiments, we finally identified three key genes: MX2, OAS1, and OAS2. The results demonstrated that these 3 genes are highly expressed in dermatomyositis and are enriched in KEGG pathways, including the cytosolic DNA signalling pathway and cytokine-cytokine receptor interaction signalling pathway. Hallmark function was enriched in interferon-*α* response and interferon-*γ* response function.

Although the exact pathogenesis of dermatomyositis has not been fully elucidated, studies have suggested that the mechanism may cause upregulation or abnormalities in transduction signalling through the interferon pathway [22, 23]. There is substantial evidence that interferons (IFNs) are considered critical in skin disease and muscle disease in patients with dermatomyositis. Increased type I IFN signalling found in skin biopsies of lesions from 16 patients with dermatomyositis [24] and skin activity in adult dermatomyositis has been shown to correlate with type I IFN gene signatures [25, 26]. IFN signalling can be used to measure disease activity in adult and adolescent subjects with dermatomyositis markers, and identification of the signal in peripheral blood samples could be an alternative to the more invasive muscle biopsy technique [27]. Epstein-Barr virus (EBV) and cytomegalovirus (CMV) belong to the human herpes virus (HHV) family, and most adults worldwide are susceptible to these viruses [28, 29]. EBV infection leads to excessive production of interferons (IFNs) by T cells, which are proinflammatory cytokines essential for systemic autoimmunity. CMV can infect several cell types, including epithelial cells, hematopoietic cells, and connective tissue [30]. EBV and CMV have been found to play a role in autoimmunity and may trigger a range of inflammatory factors that can exacerbate immune system disorders [31]. Although the pathogenesis of dermatomyositis is currently unclear, an immune imbalance is thought to be central to disease progression.

As an inhibitor of interferon (IFN) induction, myxovirus resistance-2 (MX2) has potent inhibitory activity against HIV-1 as well as herpes and hepatitis B viruses [32, 33]. Expression of MX2 reduces permissibility to various lentiviruses, and knockdown of MX2 expression using RNA interference has been shown to reduce IFN-*α* anti-HIV-1 potency [34]. It has been shown that MX2 is a cell autonomous anti-HIV-1 resistance factor whose purposeful mobilization may serve as a novel approach for the treatment of HIV/AIDS [35]. In the present study, MX2 is also expected to be a potential target for dermatomyositis treatment.

Interferon- (IFN-) induced double-stranded RNA activating enzymes are the so-called OAS proteins. The OAS patients includes 4 members: OAS1, OAS2, OAS3, and OASL [36]. Expression of the OAS gene family is highly regulated in patients with juvenile dermatomyositis, similar to the immune response to dsRNA virus infection [37]. Several studies have suggested that excessively keratinised cells may be responsible for the development of skin lesions in patients with dermatomyositis, in which OAS genes may activate one of the apoptotic cell death mechanisms [38] and resulted that the OAS/RNaseL pathway is a new effector of BRCA1 and IFN-*γ*-mediated apoptosis [39].

The present study has some drawbacks, as there is a lack of follow-up wet experiments to verify the mechanisms of the 3 genes identified to strengthen the results, in addition to the fact that the impact of the 3 genes on clinical prognosis has not yet been studied. This should be analysed in future studies in the context of clinical samples and survival.

In conclusion, a total of 3 genes associated with the development of dermatomyositis—MX2, OAS1, and OAS2—were identified in this study through a series of information biology analyses, which are expected to be biomarkers or drug targets to some extent for the diagnosis of dermatomyositis. Further studies are needed to elucidate the related mechanisms.

## Figures and Tables

**Table 1:** Top 5 MCODE pathway and process enrichment analysis for each group.

MCODE	GO	Description	Log_10_ (*p*)
MCODE 1	R-HSA-195258	RHO GTPase effectors	−12.0
MCODE 1	R-HSA-9664422	FCGR3A-mediated phagocytosis	−10.4
MCODE 1	R-HSA-9664417	Leishmania phagocytosis	−10.4
MCODE 2	R-HSA-72163	mRNA splicing—major pathway	−11.4
MCODE 2	R-HSA-72172	mRNA splicing	−11.3
MCODE 2	R-HSA-72203	Processing of capped intron-containing pre-mRNA	−10.4
MCODE 3	GO:0040029	Regulation of gene expression, epigenetic	−6.1
MCODE 3	GO:0051052	Regulation of the DNA metabolic process	−6.0
MCODE 3	GO:0034728	Nucleosome organization	−5.8
MCODE 4	R-HSA-909733	Interferon alpha/beta-signalling	−37.1
MCODE 4	R-HSA-913531	Interferon signalling	−30.3
MCODE 4	R-HSA-1280215	Cytokine signalling in the immune system	−22.4
MCODE 5	R-HSA-5696395	Formation of incision complex in GG-NER	−10.9
MCODE 5	R-HSA-5696399	Global genome nucleotide excision repair (GG-NER)	−9.4
MCODE 5	R-HSA-5696398	Nucleotide excision repair	−8.8

## Data Availability

The data used to support the findings of this study are included within the article.

## Additional Points

First time to explore the potential pathogenesis of dermatomyositis through the information biology analysis of gene chip. First time to identify diagnostic biomarkers and biological pathways in dermatomyositis based on WGCNA.
